# The activity of *engrailed* imaginal disc enhancers is modulated epigenetically by chromatin and autoregulation

**DOI:** 10.1371/journal.pgen.1010826

**Published:** 2023-11-15

**Authors:** Yuzhong Cheng, Fountane Chan, Judith A. Kassis

**Affiliations:** Division of Developmental Biology, *Eunice Kennedy Shriver* National Institute of Child Health and Human Development, National Institutes of Health, Bethesda, Maryland, United States of America; Geisel School of Medicine at Dartmouth, UNITED STATES

## Abstract

*engrailed* (*en*) encodes a homeodomain transcription factor crucial for the proper development of Drosophila embryos and adults. Like many developmental transcription factors, *en* expression is regulated by many enhancers, some of overlapping function, that drive expression in spatially and temporally restricted patterns. The *en* embryonic enhancers are located in discrete DNA fragments that can function correctly in small reporter transgenes. In contrast, the *en* imaginal disc enhancers (IDEs) do not function correctly in small reporter transgenes. En is expressed in the posterior compartment of wing imaginal discs; in contrast, small IDE-reporter transgenes are expressed mainly in the anterior compartment. We found that En binds to the IDEs and suggest that it may directly repress IDE function and modulate En expression levels. We identified two *en* IDEs, O and S. Deletion of either of these IDEs from a 79kb HA-en rescue transgene (*HAen79*) caused a loss-of-function *en* phenotype when the *HAen79* transgene was the sole source of En. In contrast, flies with a deletion of the same IDEs from an endogenous *en* gene had no phenotype, suggesting a resiliency not seen in the *HAen79* rescue transgene. Inserting a gypsy insulator in *HAen79* between *en* regulatory DNA and flanking sequences strengthened the activity of *HAen79*, giving better function in both the ON and OFF transcriptional states. Altogether our data suggest that the *en* IDEs stimulate expression in the entire imaginal disc, and that the ON/OFF state is set by epigenetic memory set by the embryonic enhancers. This epigenetic regulation is similar to that of the *Ultrabithorax* IDEs and we suggest that the activity of late-acting enhancers in other genes may be similarly regulated.

## Author summary

Genes that control development are often used at different times and places in a developing embryo. Transcription of these important genes must be tightly regulated; therefore, these genes often have large arrays of regulatory DNA. In Drosophila, discrete fragments of DNA (enhancers) can be identified that turn genes on in patterns in the early embryo. In cells where the genes are transcriptionally ON, there are active modifications on chromatin, setting later enhancers in a transcription-permissive environment. In cells where the genes are OFF, repressive chromatin marks keep later enhancers inactive. In this paper we studied two late enhancers of the Drosophila *en* gene. We show that the correct activity of these enhancers is dependent on being next to other, earlier acting *en* enhancers. Our data also show that En can repress its own expression, likely directly by acting on these late enhancers. The chromatin-regulated activity of these *en* late enhancers is similar to what was described for a late enhancer of another Drosophila developmental gene, *Ubx*. We suggest that this mode of regulation is likely to be important for many late-acting developmental enhancers in many different organisms.

## Introduction

Developmentally important transcription factors are expressed in spatially and temporally restricted patterns in the precursors of many different cell types. These complex gene expression patterns are generated by a large number of enhancers, traditionally defined by their abilities to stimulate patterned gene expression in transgenes (reviewed in [1]). Many developmental genes have so-called “shadow enhancers”; that is, more than one enhancer that can drive transcription in a similar pattern. Enhancers with overlapping functions are thought to impart robustness to transcription of these important genes [2–5]. In addition to pattern setting enhancers (which contain binding sites for both transcriptional activators and repressors [1]), developmental genes are regulated by the Polycomb (PcG) and Trithorax group genes (TrxG). Studies in Drosophila show that PcG and TrxG genes can impart a memory of the early pattern by setting the chromatin in an ON or OFF transcriptional state (reviewed in [6,7]). We are interested in how chromatin environment influences the enhancer activity of developmental genes.

**Fig 1:**
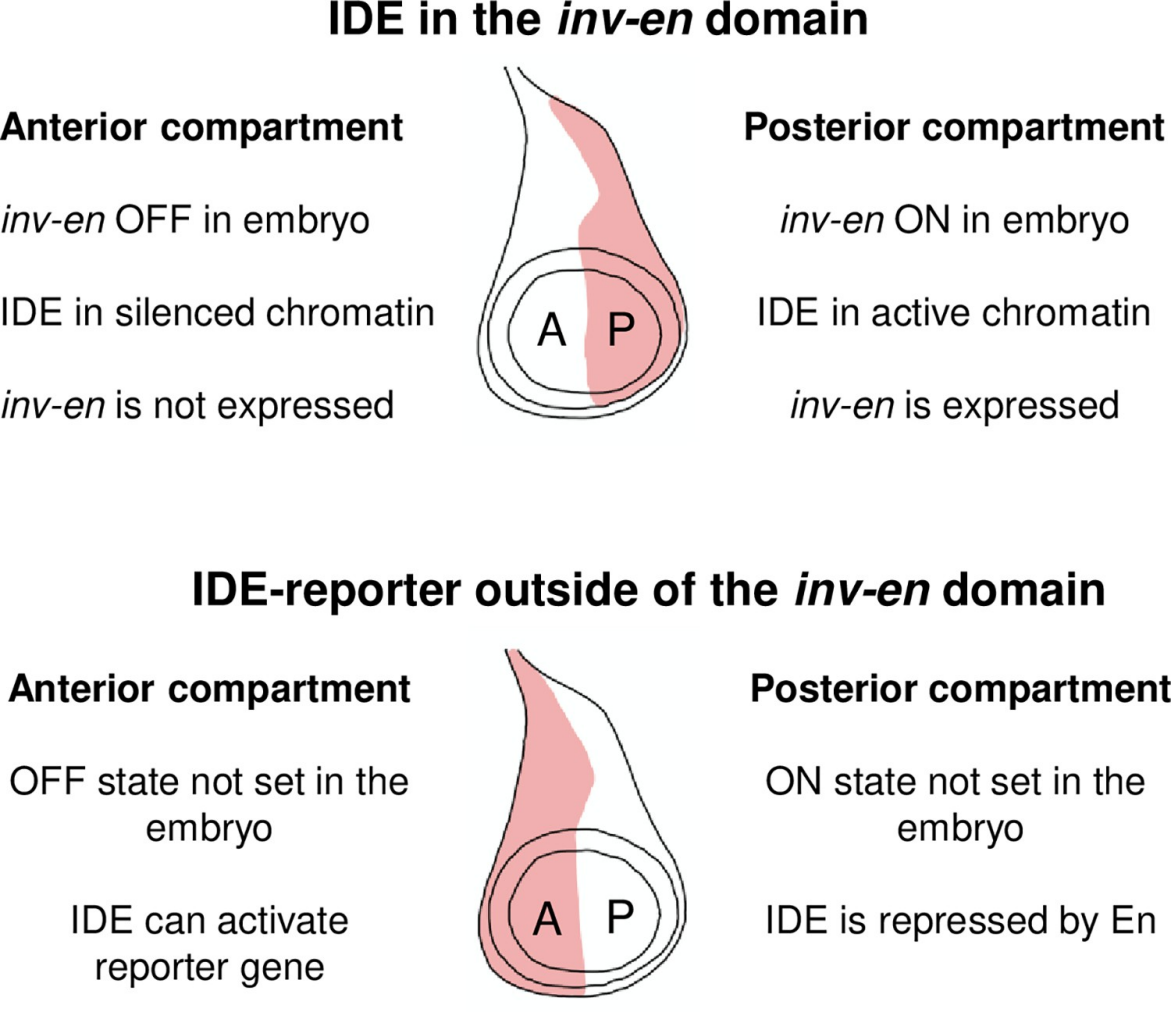
Model of how the *en* imaginal disc enhancers (IDEs) function inside and outside the *inv-en* domain. Diagrams of a wing disc with expression (red shading) in either the posterior (P) or anterior (A) compartment are shown.

The Drosophila *engrailed* (*en*) gene encodes a homeodomain transcription factor whose best-known functions are in embryonic segmentation and specification of the posterior compartment in larval imaginal discs, precursors of the external structures of the adult [8–10]. En is expressed in the embryo in a series of stripes in the ectoderm, and subsets of cells in the central and peripheral nervous systems, hindgut, fat body, posterior spiracles, and head [11]. Using a reporter gene in transgenic flies, we identified 20 embryonic enhancers spread over a 66kb region including DNA upstream, within, and downstream of the 4kb *en* transcription unit [12]. However, we were unable to identify a fragment of DNA that drove expression of a reporter gene in the posterior compartment of imaginal discs in an *en*-like pattern. We speculated that, like the imaginal disc enhancers of the *Ultrabithorax* (*Ubx*) gene [13–16], the ‘ON-OFF’ state of the *en* imaginal disc enhancers is set by the embryonic expression pattern and remembered throughout development through epigenetic memory; without this epigenetic memory, the *en* imaginal disc enhancers could not regulate a reporter gene in the appropriate pattern (Fig 1).

*en* exists in a gene complex with *invected* (*inv*). *inv* encodes a closely related homeodomain protein that is largely co-regulated with *en* [17,18]. In the ‘OFF’ transcriptional state, H3K27me3, the repressive chromatin mark put on by the Polycomb protein complex PRC2, covers the entire *inv-en* domain, showing that *inv-en* is a target for Polycomb-mediated repression ([19,20]. Consistent with this, Polycomb group genes (PcG) are required to silence *inv-en* expression where they are not normally expressed in embryos and imaginal discs [21–24]. In our dissection of *inv-en* regulatory DNA we found two fragments of DNA that acted as enhancers of reporter genes in imaginal discs [12] but, unexpectedly, the reporter genes were expressed more strongly in the anterior compartment, the opposite of where En is expressed. Previous studies showed that overexpression of En via an inducible transgene can silence En expression in imaginal discs [25,26]. We hypothesized that when the *en* IDEs were outside of the *inv-en* domain they 1) were not silenced in the anterior compartment by PcG repressive marks and 2) were not covered by active chromatin marks in the posterior compartment and were susceptible to repression by En (Fig 1).

Here we study the activity of two *en* IDEs using three approaches 1) testing their activities in small transgenes 2) deleting them from *HAen79*, a 79kb transgene with HA-tagged En, that can rescue *inv-en* double mutants [27], and 3) deleting them from *invΔ33*, a chromosome that contains a 33kb deletion of *inv* DNA, creating a mimic at the endogenous *en* locus of the sequences present in *HAen79* (called *en80* in [27]). Our results suggest that the En protein directly represses its own expression through the imaginal disc enhancers and other sequences within the *inv-en* domain. Deletion of either imaginal disc enhancer from the *HAen79* transgene causes a loss-of-function *en* phenotype, showing that these fragments are IDEs for *en*. In contrast, the same deletions do not cause phenotypes when deleted from the *invΔ33* endogenous locus. Altogether our experiments show that the function of the imaginal disc enhancers is regulated by the chromatin environment of the endogenous *inv-en* domain.

## Results

**Fig 2:**
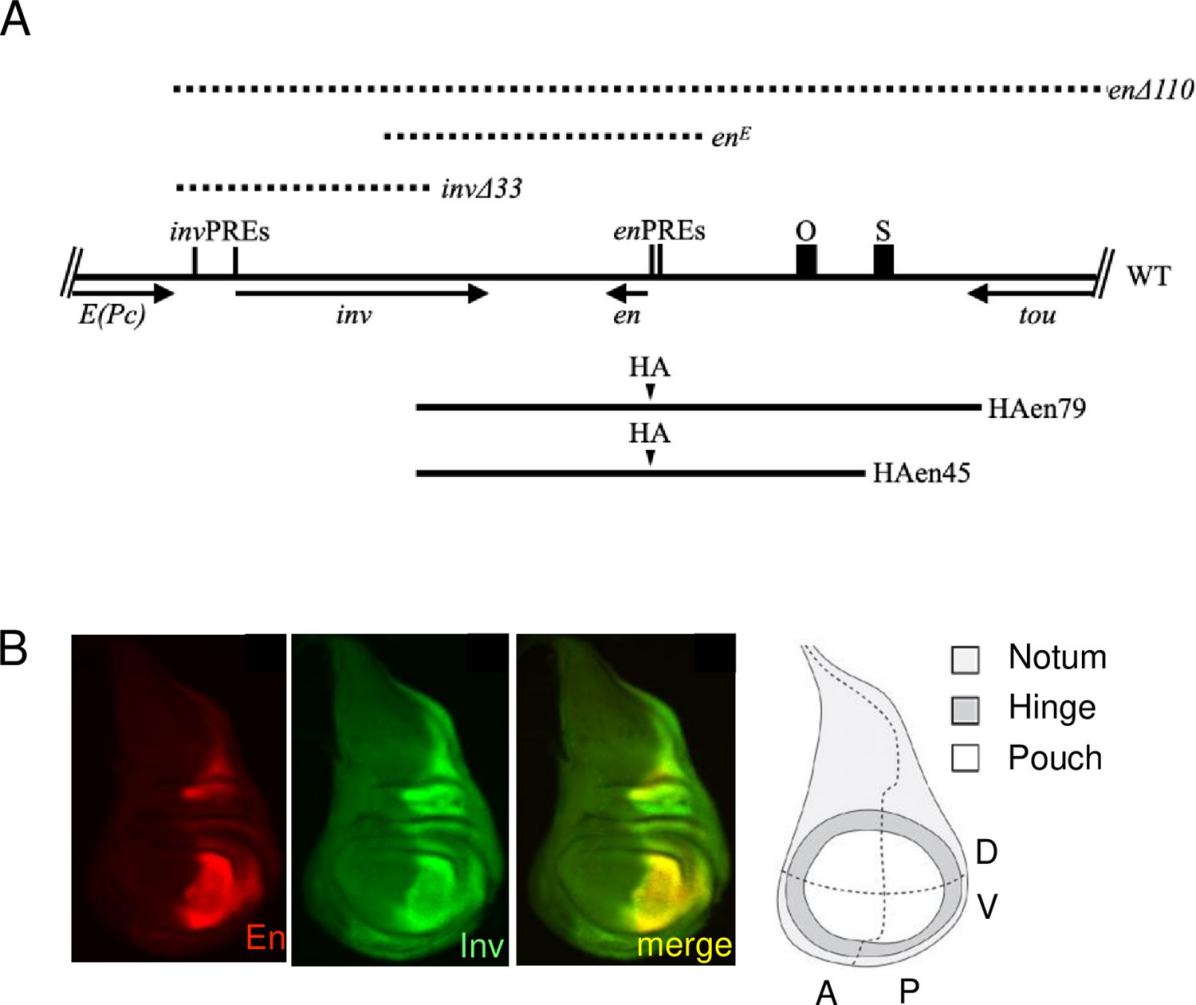
Map of *invΔ33*, transgenes, PREs and imaginal disc enhancers. (A) Diagram of the *inv-en* region of the genome with flanking genes. The black boxes labeled O and S are the locations of the IDEs studied in this paper. Vertical lines show the locations of the constitutive *inv* and *en* PREs. The arrows denote the direction and extent of the transcription units. The DNA deleted in *invΔ33*, *enΔ110*, and *en*^*E*^ is shown by dotted lines. Bottom, the extent of the DNA present in the two large transgenes used in this study is shown by black lines. In these transgenes, En is labeled on the N-terminus with a single HA-tag [12]. (B) Expression pattern of En and Inv in a wild-type wing disc. A fate map of a wing imaginal disc is shown on the right. A-anterior, P-posterior, D-dorsal, V-ventral. Diagram is from [46].

The *inv* and *en* genes are contained within a 113kb domain flanked by the genes *E(Pc)* and *tou* (Fig 2A). *en* is required for both embryonic and adult development. In contrast, the *inv* gene is not required for viability or fertility in the laboratory [18]. In many experiments in this paper, we use either a large transgene (*HAen79*) or a mutated *inv-en* domain (*invΔ33*) that encode no Inv protein to study the function of the imaginal disc enhancer (IDE) (Fig 2A). Table 1 contains a list of the transgenes and *inv-en* mutants used in our experiments. Inv and En are co-expressed in embryos and imaginal discs (Fig 2B) [12,18]. In some experiments with transgenes, we examined Inv expression from the wildtype *inv-en* domain in order to compare expression of the endogenous locus with the HA-en transgene (see below).

**Table 1:** Transgenes and *inv-en* mutants used in this paper.

**Small transgenes**	**Vector**	**En DNA Fragment Coordinates** ^**2**^	
O-H-enlacZ	*H-P[en]* ^1^	O-7435274-7439183; H-7415785-7423711	
S-H-enlacZ	*H-P[en]*	S-7448809-7451645; H-7415785-7423711	
O-enlacZ	*P[en]*	O-7435274-7439183	
S-gal4	*pBPGUw*	S-7448809-7451645	
SS2-gal4	*pBPGUw*	SS2-7448809-7450141	
SS1-gal4	*pBPGUw*	SS1-7450142-7451645	
**Large transgenes**	**En DNA Coordinates**	**Reference**	
*HAen45*	7404008–7448931	[12]	
*HAen79*	7386838–7466000	[12]	
**Modified *HAen79***	**Modification**	**Method**	
*HAen79stop* ^3^	stop codons inserted in *en*	Recombineering/transgene insertion	
*HAen79ΔO*	Fragment O deleted	Recombineering/transgene insertion	
*HAen79ΔS*	Fragment S deleted	Recombineering/transgene insertion	
*HAen79ΔSS2*	Fragment SS2 deleted	Recombineering/transgene insertion	
*HAen79GyW*	Gypsy Element added at MW side	CRISPR/Cas9 of *HAen79* flies	
*HAen79GyB*	Gypsy Element both sides	CRISPR/Cas9 of *HAen79GyW* flies	
**Genomic mimic of *HAen79***	**Deleted sequences**	**Method**	**Reference**
*invΔ33*	7353743–7386877	CRISPR/Cas9	[27]
**CRISPR/Cas9 modifications of *invΔ33***	**Modification**		
*invΔ33ΔO*	Fragment O deleted from *invΔ33*		
*invΔ33ΔS*	Fragment S deleted from *invΔ33*		
*invΔ33ΔSS2*	Fragment SS2 deleted from *invΔ33*		
*invΔ33ΔOΔSS2*	Fragment SS2 deleted from *invΔ33ΔO*		
*invΔ33HAenΔSS2*	HA-tag added to En on *invΔ33ΔSS2*		
*HAen*	HA-tag added to En on a wildtype chromosome		
***inv-en* deletions**	**Deleted coordinates (size)**	**Generated using**	**Reference**
*enΔ110*	7353743–7463977 (110kb)	CRISPR	[27]
*en* ^*E*^	7383679–7425016 (41.3kb)	P-element excision	[18]
*en* ^*X31*^	7332587–7536107 (203.5kb)	X-rays	[10]

^1^*P[en]* contains the *en* promoter, 396bp of upstream sequences, and an untranslated leader fusion between *en* and an Adh-Reporter gene [12]. Fragment H contains 7.9kb of regulatory sequences from -396bp to

-7.9kb including enhancers for embryonic stripes but no disc enhancers [12].

^2^All coordinates are on chromosome 2R, Genome Release v5.

^3^[27]

### Fragments O and S are imaginal disc enhancers

The locations of two fragments of DNA, O and S, that drove reporter gene expression mainly in the anterior compartment of imaginal discs are shown in Fig 2 [12]. To test the hypothesis that the ‘ON-OFF’ state of these enhancers could be set at the embryonic stage, we cloned them in a vector that gives striped expression throughout most of embryogenesis but no expression in imaginal discs (construct H [12], S1 Fig). Fragment O is 3.9kb and includes some stripe enhancers for early and mid-embryogenesis but not late embryogenesis [12]. For S, we used a 2.8kb fragment, considerably smaller than the 6.7kb fragment we previously studied [12]. The coordinates of this fragment were set by an overlap of our original S fragment and an imaginal disc enhancer identified in a screen of genomic fragments for cis-regulatory activity in imaginal discs (line GMR94D09, [28]). There are no embryonic enhancers present in this 2.8kb fragment S. Construct H contains both stripe enhancers and Polycomb response elements (PREs) that might impart transcriptional memory on the O or S IDEs leading to expression of the reporter gene, ß-galactosidase (ßgal), in the posterior compartment of wing discs. For S, this did not occur. The expression of ßgal in three independent insertion lines was stronger in the anterior than the posterior compartment of the wing disc (S1 Fig). ßgal expression from the O construct was quite variable. In one line, ßgal was OFF in the anterior compartment, like En, but only partially ON in the posterior compartment (S1 Fig). In another, ßgal was expressed in the posterior compartment, and mostly silenced in the anterior, and in another, anterior expression was stronger than posterior, similar to expression driven by S in this vector. This variability in expression pattern illustrates the strong influence of chromatin environment on the activity of these IDEs. Nevertheless, these results confirmed that these fragments could act as IDEs in another reporter vector. Finally, although in this paper we describe the activity of S and O in wing discs, both these enhancers also drive expression in all other discs examined (haltere, leg, and eye-antennal discs, S2 Fig).

**Fig 3:**
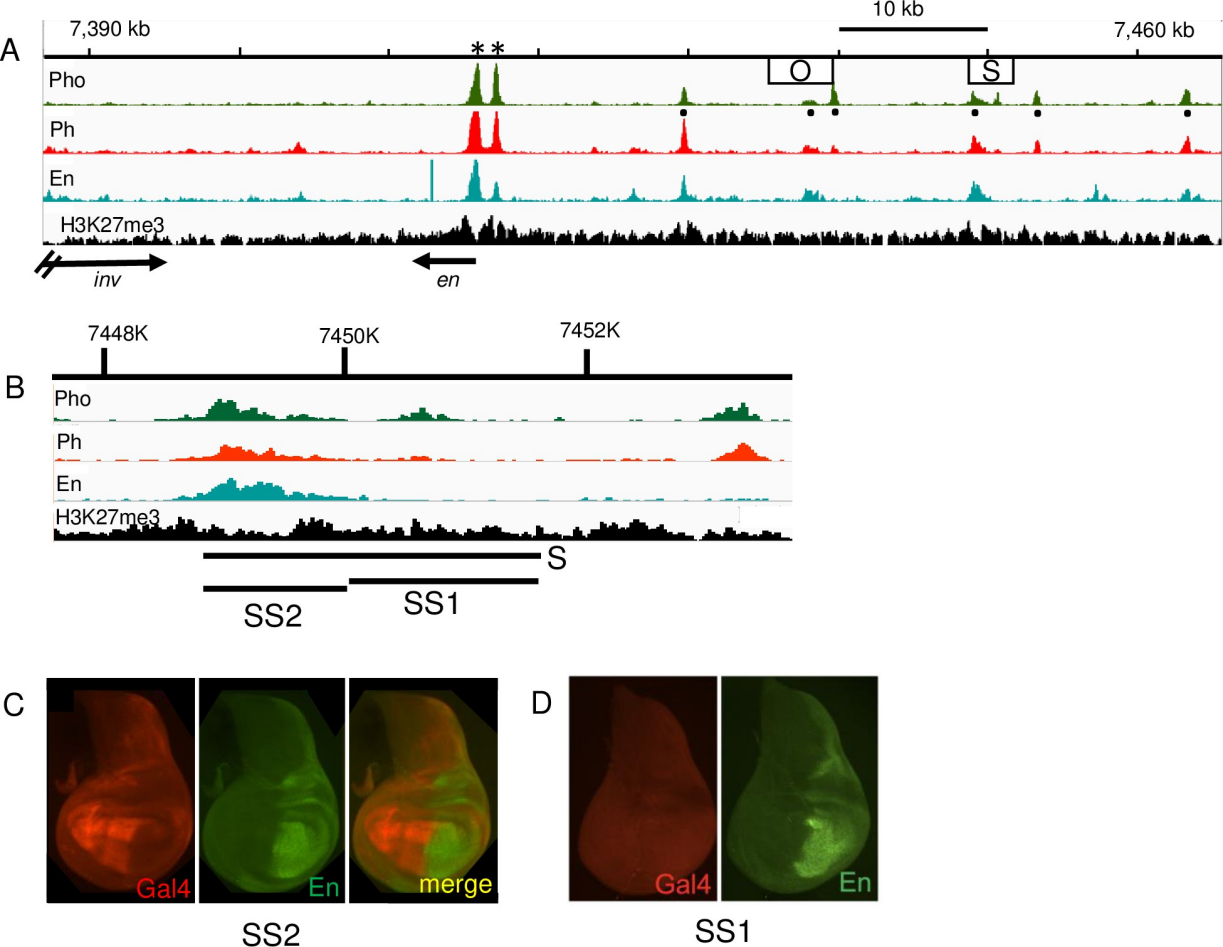
Fragment S binds En and stimulates expression of a reporter gene in the anterior compartment in a small transgene. (A) ChIP-seq data on 3^rd^ instar larval brains and discs for Pho, Ph, En, and H3K27me3 over the genomic region present in *invΔ33* (Coordinates chr2R, version dm5; sequences from GSE76892 [47]). Astericks indicate the position of the constitutive (aka major) *en* PREs. “Minor” or tissue specific PREs are marked by black dots below the Pho ChIP-seq peaks [20]. These could be dual function elements that serve as enhancers or silencers dependent on the context [48]. Locations of the O and S fragments are shown as boxes. (B) Expanded view of the region around fragment S showing the locations of the SS2 and SS1 fragments. (C,D) Gal4 (red) expression in wing discs from transgenic flies containing SS1 or SS2 cloned in front of Gal4 (in *pBPGUw*, [42]). En (green) is shown for comparison. These transgenes were inserted at attP40. Similar results were obtained with the same transgenes inserted at attP2. At least 10 discs were examined for each genotype and a representative disc is shown.

We cloned the S fragment into a different vector used to detect enhancer activity and dissected it into two smaller fragments, SS2 and SS1 (Fig 3). Chromatin-immunoprecipitation followed by sequencing (ChIP-seq) in 3^rd^ instar larval brains and discs show the location of the Polycomb proteins Pho, Ph, and the En protein and the H3K27me3 chromatin mark over the DNA present in the *invΔ33* allele (Fig 3A). Normally, En expression is silenced by Polycomb proteins in the anterior compartment in discs [21,23,24], consistent with H3K27me3 covering this region of the chromosome in this mixed cell population. S, SS2, and SS1 were cloned in front of the GAL4 reporter gene (Figs 2D and 3C) and integrated into two different insertion sites: attP40 and attP2. At both chromosomal locations, S and SS2 gave nearly identical expression patterns, in the anterior compartment. There is an En ChIP-seq peak directly over the SS2 fragment (Fig 3B). En contains an active repression domain [29], overexpression of En by an inducible transgene silences *en* expression [25,26]. We suggest that En may directly repress the expression of the transgene by binding to the S enhancer. SS1 has no enhancer activity in wing discs (Fig 3D).

We next turned our attention to two large HA-en transgenes, one 45kb and one 79kb (Fig 2A). We previously showed that *HAen45* can rescue *en* mutants, but not double mutants of *inv* and *en* [12]. *HAen79* transgene rescues *inv-en* double mutants, including *enΔ110* that deletes the most of the *inv-en* domain [12,27]. Despite this, the expression of HA-en from these transgenes is not normal in wing discs in a wildtype background (S3 Fig). In *HAen45*, HA-en is nearly absent in the pouch region of the wing disc at three different attP insertion sites and variegated at the other (S3A and S3E Fig). Expression of HA-en in the *HAen45* wing pouch is partially restored in the presence of the *en*^*B86*^ mutation, a deletion of 53bp in the coding region of En, that produces no detectable En protein [18] (S3B Fig). These data suggest that the repression of *HAen45* is mediated by the En protein itself and not via an interaction of the transgene with the endogenous *inv-en* gene, as has been seen at the Drosophila *spineless* gene [30]. *HAen79* is expressed better than *HAen45*, but there are still regions of the wing disc where it is not expressed (S3C and S3E Fig). The size of the repressed region is variable from disc to disc, and the expression is variegated at two different insertion sites (S3 Fig). We suggest that this variegated expression is the result of unstable gene expression, a competition between the ON and OFF transcription states set by epigenetic marks.

We also asked whether the HA-en made by the transgene is necessary for its variegated expression. We made *HAen79-stop* that contains a stop codon in En and produces a non-functional En protein [27]. Like *HAen79@attP40*, *HAen79-stop@attP40* is expressed in only part of the wing pouch (S3F Fig) and its expression is variegated. We conclude that the *HAen79* transgene is repressed by En expressed from the wildtype *inv-en* domain and that the HA-en protein contributes very little to this repression.

**Fig 4:**
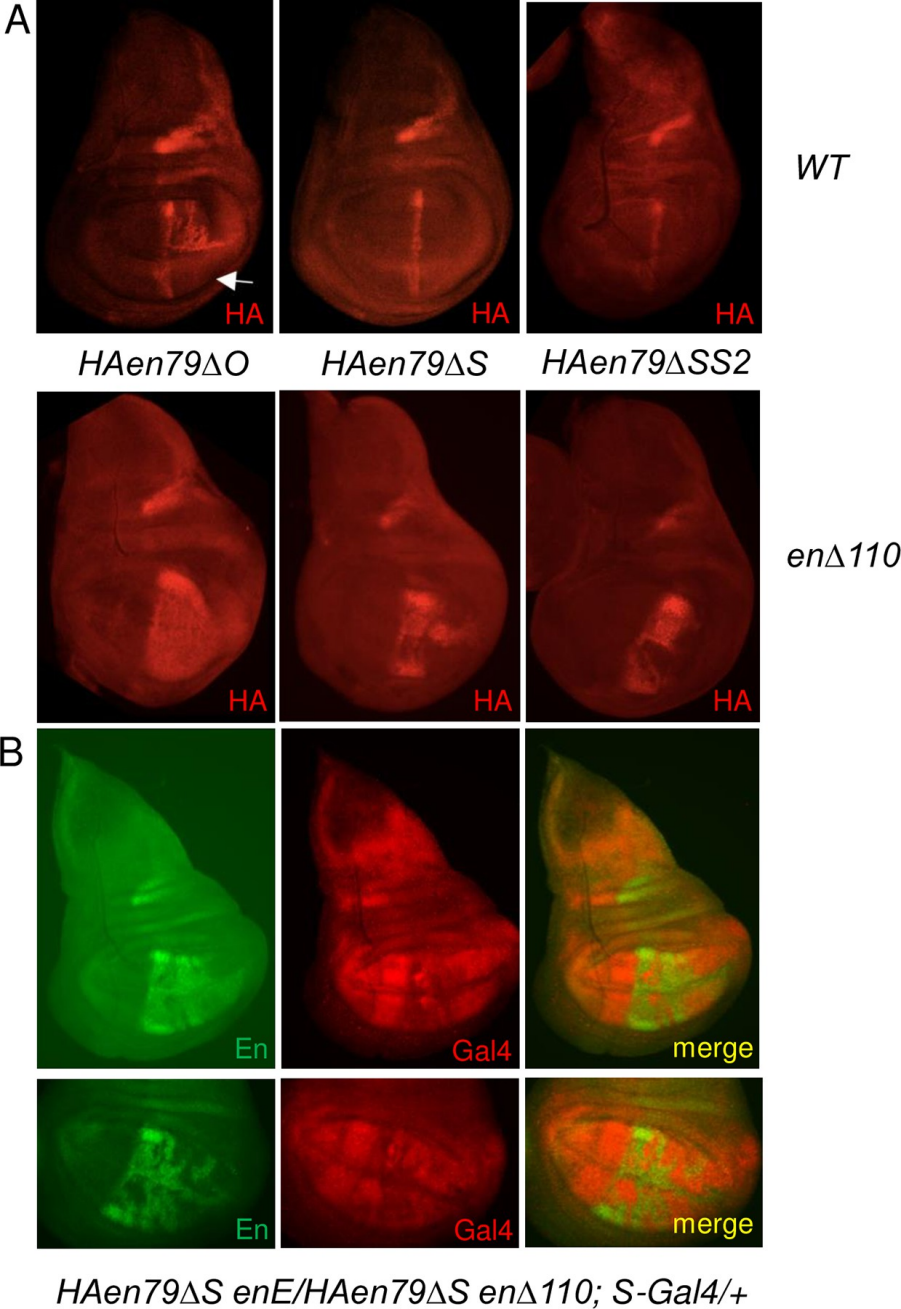
En represses expression of fragment O and S containing transgenes. (A) Top row: HA expression from *HAen79ΔO*, *HAen79ΔS*, and *HAen79ΔSS2* (all inserted at attP40) on a wildtype chromosome. Bottom: The same transgenes on an *enΔ110* chromosome. All discs are homozygous for the indicated genotype. Arrow points to the ventral portion of the wing disc. (B) En (green) and Gal4 (red) in two different wing imaginal discs of the genotypes *HAen79ΔS enE/HAen79ΔS enΔ110; S-gal4/+*. The bottom row shows a close-up of the pouch region of a different wing disc. *HAen79ΔS* is the only source of En in this genotype and is expressed in a variegated manner in the wing pouch. S-gal 4 is expressed in the wing pouch predominantly in cells that do not express En. At least 10 discs were examined for each genotype and a representative disc is shown.

We next deleted fragments O, S, or SS2 from the *HAen79* transgene, inserted them at *attP40*, and compared the expression of HA-en in wing imaginal discs, both with and without wildtype Inv and En (Fig 4). Deleting fragment O decreases expression of HA-en in a wildtype background, especially in the ventral region of the wing pouch (Fig 4A, white arrow). The O fragment contains an embryonic stripe enhancer for the ventral part of the embryo at stage 12 of development [12]. Our data suggest either that O also contains an enhancer for the ventral wing disc, or that epigenetic memory is impaired when this embryonic enhancer is removed. Deleting fragments S or SS2 from *HAen79* gave essentially the same result in wing discs (Fig 4A). In a wildtype background, expression is only observed in a line at the anterior-posterior (A-P) boundary. There are three different enhancers for this A-P boundary line present in *HAen79*, and they are not within fragments O or S [12]. Removal of the *inv-en* domain leads to almost wildtype expression from *HAen79ΔO* and more, but still variegated, expression of *HAen79ΔS* or *HAen79ΔSS2* (Fig 4A). The wing phenotypes of *HAen79ΔO*, *ΔS* or *ΔSS2 enΔ110* are consistent with these expression patterns (S4A Fig). Minor wing vein defects are seen in *HAen79ΔO enΔ110* wings, and more severe phenotypes are seen in *HAen79ΔS enΔ110* and *HAen79 ΔSS2enΔ110* wings, including the presence of anterior-like bristles on the posterior wing margin, indicating a posterior-to-anterior transformation (S4A Fig). Altogether these data show that fragments O, S, and SS2 contain IDEs.

### A strong correlation between En protein and repression of the S enhancer

We used the variegated expression of HA-en from *HAen79ΔS* as a tool to address the correlation between En expression and repression of the S enhancer. We constructed a genotype *HAen79ΔS enΔ110/ HAen79ΔS en*^*E*^*; S-Gal4@attP2/+* and examined En and Gal4 distribution in wing discs. *en*^*E*^ is a 41kb deletion that removes En and produces a truncated Inv protein that lacks the homeodomain (Fig 2A). In this background, the only source of En is from the *HAen79ΔS* transgene. Strikingly, in the posterior compartment of the wing pouch, *S-Gal4* is repressed in the cells where En is expressed (Fig 4B). These data, along with ChIP data that show En binding to S, support the hypothesis that En can directly repress the S-enhancer in the wing pouch.

### One copy of the *HAen79* transgene is haploinsufficient

Flies survive well with one copy of the *inv-en* domain and have no known phenotypes. That is not the case with the *HAen79* transgenes. While homozygous *HAen79 enΔ110* flies survive with only minor wing defects, flies with only one copy of *HAen79* in a homozygous *enΔ110* background have wing defects and survive poorly (S4 Fig and Table 2). Some *HAen79 enΔ110/enΔ110* flies hatch and then stick to the sides of the vial or fall in the food and die, suggesting a defect in nervous system development. In contrast, *HAen79ΔO* or *HAen79ΔS enΔ110/*three different *inv-en* deletions (*enΔ110*, *enE* and *enX31*) die as pharate adults with severe leg defects, and wings that are usually not expanded (S4C Fig). A rare *HAenΔO enΔ110/en*^*E*^ fly with an expanded wing showed a lack of wing veins throughout most of the wing and deformed legs (S4C Fig). *HAen79ΔSS2 enΔ110/inv-en* deletion flies survive with wing defects similar to those seen in *HAen79ΔSS2 enΔ110* homozygotes and have no leg defects. These data suggest that ΔS takes out more regulatory sequences than does ΔSS2. Thus, although the *HAen79* transgene can rescue *inv-en* double mutants, it is a not equivalent to a wildtype *en* locus. Deleting either the O or S fragment from the *HAen79* transgene leads to leg defects when these transgenes are the only source of En (S4 Fig). This provides further evidence that these fragments are also enhancers in leg discs.

**Table 2:** Viability and phenotype of flies with a single copy of the *en* gene.

Genotype of fathers	Genotype of mothers								
	*CyO / enΔ110*			*CyO / en* ^*E*^			*CyO / enX31*		
	Progeny			Progeny			Progeny		
	*CyO*	*enΔ110*		*CyO*	*en* ^*E*^		*CyO*	*enX31*	
	#	#	Phenotype	#	#	Phenotype	#	#	Phenotype
*invΔ33*	68	77	+	51	34	+	63	36	WO
*invΔ33ΔO*	46	55	WO	48	46	WO	84	46	WO
*invΔ33ΔS*	37	41	WO	68	77	+	106	44	WO
*invΔ33ΔSS2*	46	47	WO	41	42	+	99	45	WO
*invΔ33ΔOΔSS2*	94	79	WO**	73	96	WO**	ND	ND	ND
*invΔ33HAenΔSS2*	44	46	WO	54	34	+	99	45	WO
*HAen*	41	59	+	48	51	+	68	35	+
*HAen79 enΔ110*	146	9*	WO**	136	2*	WO**	80	2*	WO**
*HAen79GyB enΔ110*	151	86	WO	110	98	WO	75	41	WO
*HAen79GyMW enΔ110*	79	98	WO	77	76	WO	93	41	WO

Mothers of the genotype on top were crossed to fathers of the genotype on the left and their progeny were counted.

See Table 1 and Fig 2 for the extent of deletions in *enΔ110*, *en*^*E*^, and *enX31*.

WO-Wings held out. The wings out phenotype is correlated with the loss of the *inv* DNA [20].

+ Wildtype

* A few flies hatch and either fall in the food and die or are stuck to the pupal case or sides of vial.

** Wing vein defects in posterior compartment (a loss of function phenotype).

### En expression from *invΔ33* is not sensitive to the loss of the O or SS2 enhancers

**Fig 5:**
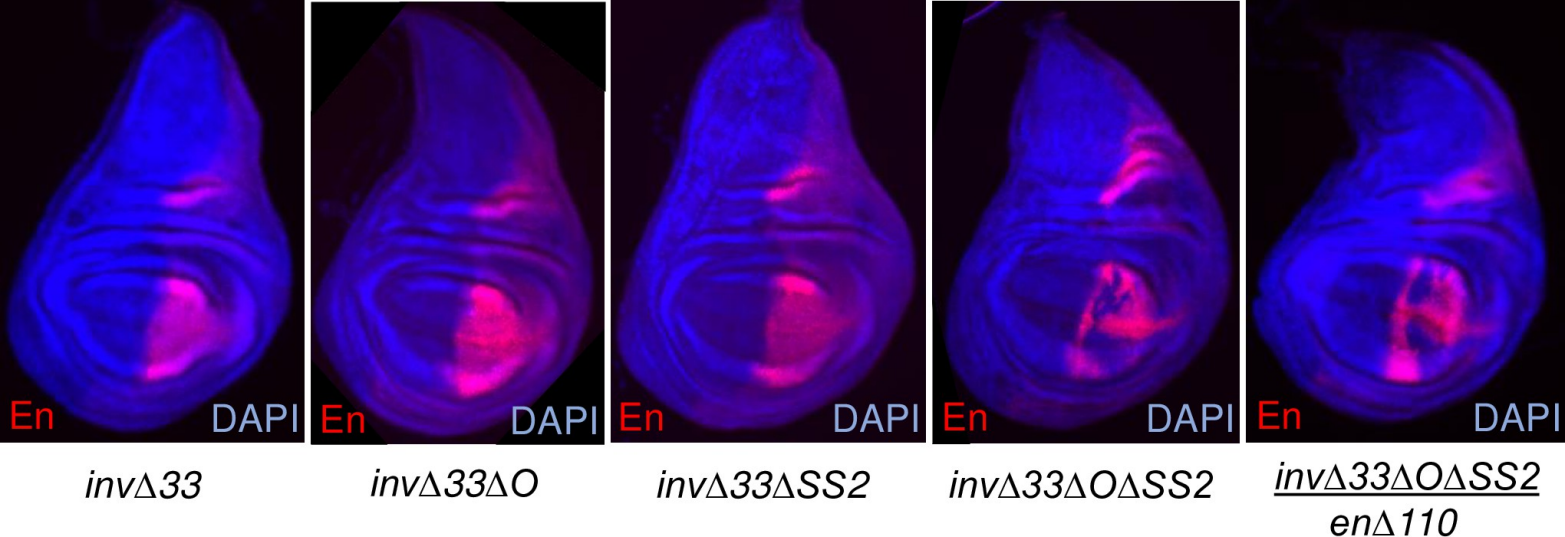
Deletions of disc enhancers from *invΔ33* reveals the stability of the endogenous locus. En in *invΔ33*, *invΔ33ΔO* and *invΔ33ΔSS2* wing imaginal discs looks like WT. En in *invΔ33ΔOΔSS2* homozygous and *invΔ33ΔOΔSS2/enΔ110* wing discs is variegated. At least 10 discs were examined for each genotype and a representative disc is shown.

*invΔ33* was created as a mimic of the *HAen79* transgene at the endogenous locus (called *en80* in [27], Fig 2A). At *invΔ33*, in addition to the 79kb present in *HAen7*9, there is 1kb of DNA just downstream of the *E(Pc)* transcription stop site. *E(Pc)* and *tou* transcription form the boundaries of the *inv-en* domain [31]. We left 1kb downstream of *E(Pc)* because we did not want to risk interfering with *E(Pc)* transcription termination. We used *CRISPR/Cas9* to delete either fragment O or SS2 from *invΔ33* and saw no difference in the En expression pattern in wing discs (Fig 5). We next tested whether *invΔ33*, *invΔ33ΔO* or *invΔ33ΔSS2* were sufficient as single copies by crossing them to three *inv-en* deletion mutants (Table 2). All three lines survive well over all the deletion mutants; none have wing vein or leg defects. Some flies hold their wings out, a phenotype associated with loss of *inv* [20]. We wondered whether adding a HA-tag to En would impair its function. We tagged En with HA on both a wildtype chromosome and on *invΔ33ΔSS2*, making *HAinvΔ33ΔSS2*. We found no evidence that the HA-tag compromised En function, as *HAinvΔ33ΔSS2* flies survive well as heterozygotes and do not have wing vein defects (Table 2). In summary, these data show that the endogenous *invΔ33* domain is resilient to the loss of a single imaginal disc enhancer.

We next deleted both fragments O and SS2 and found that En expression is variegated both in *invΔ33ΔOΔSS2* homozygotes and *invΔ33ΔOΔSS2/enΔ110* wing discs (Fig 5). This variegated expression is consistent with the hypothesis that these enhancers are regulated by chromatin modifications. *invΔ33ΔOΔSS2* survive well as heterozygotes (Table 2). Consistent with the variegated expression patterns, some wings have vein defects, whereas others do not.

We hypothesized that the endogenous locus was more stable to enhancer deletions than the HA-en transgene because it has boundaries that stabilize the chromatin state of the locus. For example, H3K27me3, the Polycomb chromatin mark, stops at the 3’ ends of the *E(Pc)* and *tou* genes at the endogenous locus [31]. On the other hand, the transgene does not have boundaries, and the H3K27me3 spreads from the *en* DNA in both directions many kilobases, stopping at actively transcribed genes [27]. We hypothesize that this destabilizes the transgene in both the ON and OFF transcriptional states, making it less stable and more sensitive to the loss of enhancers.

### Adding a gypsy element boundary to *HAen79* improves its function

**Fig 6:**
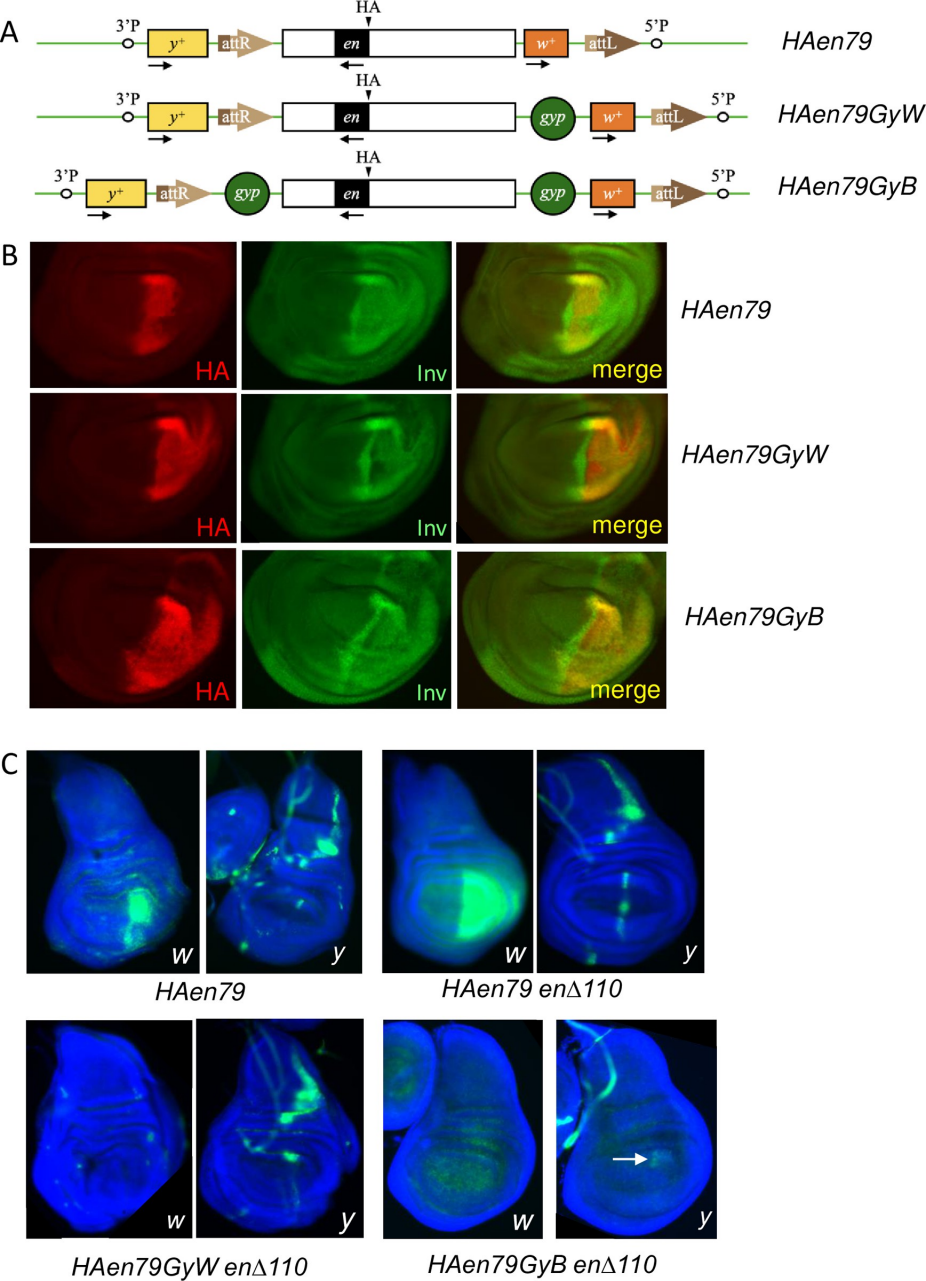
Flanking *HAen79* by gypsy elements stabilizes HA-en expression and restricts expression of flanking genes. (A) Diagram of the *HAen79* transgene and flanking sequences (not to scale). (B) HA and Inv expression in wing disc pouch from the genotypes indicated (on a wildtype chromosome). Note the expanded HA expression and variegated Inv expression in *HAen79GyW* and *HAen79GyB* compared to *HAen79*. (C) RNA in situ hybridization. Note that both the mini-*white* (*w*) and *yellow* (*y*) genes are transcribed at higher levels in the wing pouch when the endogenous *inv-en* domain is deleted (*HAen79 enΔ110*). Adding a gypsy element on the mini-*white* site completely blocks *w* expression. A small amount of *y* expression remains (white arrow) in *HAen79GyB enΔ110*. For immunostaining (B), at least 10 discs were examined for each genotype; for RNA-FISH (C), at least 7 discs were examined for each genotype.

The *HAen79* transgene also contains a mini-*white* (*w+*) gene as a marker to detect integration events into an *attP* landing site [32]. A mini-*yellow* (*y+*) gene is present at the landing site and is located downstream of the *en* transcription unit after integration of the *HAen79* into the genome (Fig 6A). We used *CRISPR/Cas9* to insert gypsy boundary elements at the ends of the 79kb HA-*en* DNA. Gypsy boundaries can block both the action of enhancers and the spreading of the H3K27me3 Polycomb mark [33–35]. Two lines were created, one with gypsy on the *w+* side, *HAen79GyW*, and the other with gypsy on both sides, *HAen79GyB*. In contrast to flies with just one copy of *en* from the *HAen79 enΔ110* chromosome, both *HAen79GyW enΔ110*, and *HAen79GyB enΔ110* flies survive well over deficiencies for *inv* and *en* and have no defects in wing veins (Table 2). Unlike the original *HAen79* line that has variegated expression in the wing pouch in the presence of a wildtype *inv-en* domain, both *HAen79GyW* and *HAen79GyB* have HA-en expression in an En-like manner in the wing pouch (Fig 6B). Unexpectedly, Inv expression is now variegated in these discs. This finding suggests that the HA-en expressed from the transgene is now able to repress the expression of Inv at the endogenous locus.

We next looked at expression of mini-*white* (*w*) and mini-*yellow* (*y*) RNA, the two genes that flank the 79kb HA-en transgene. In a wildtype background, *w* is expressed in a variegated manner in the wing pouch, like HA-en (Fig 6C). *y* is expressed mainly in the notum part of the wing disc. When the *HAen79* transgene is the only *en* gene in the genome (*HAen79 enΔ110*), *w* is expressed in the entire wing pouch, while *y* is expressed in a line at the anterior-posterior boundary in addition to the notum (Fig 6C). Inserting a gypsy element between the *en* DNA and *w* causes a complete loss of *w* expression, showing that gypsy is sufficient to block *en* enhancers from stimulating *w* expression. *y* is expressed in the notum and weakly at the A/P boundary in the wing pouch in *HAen79GyW enΔ110* wing discs. Inserting gypsy elements on both sides blocks *w* expression, and almost completely blocks *y* expression (Fig 6C).

**Fig 7:**
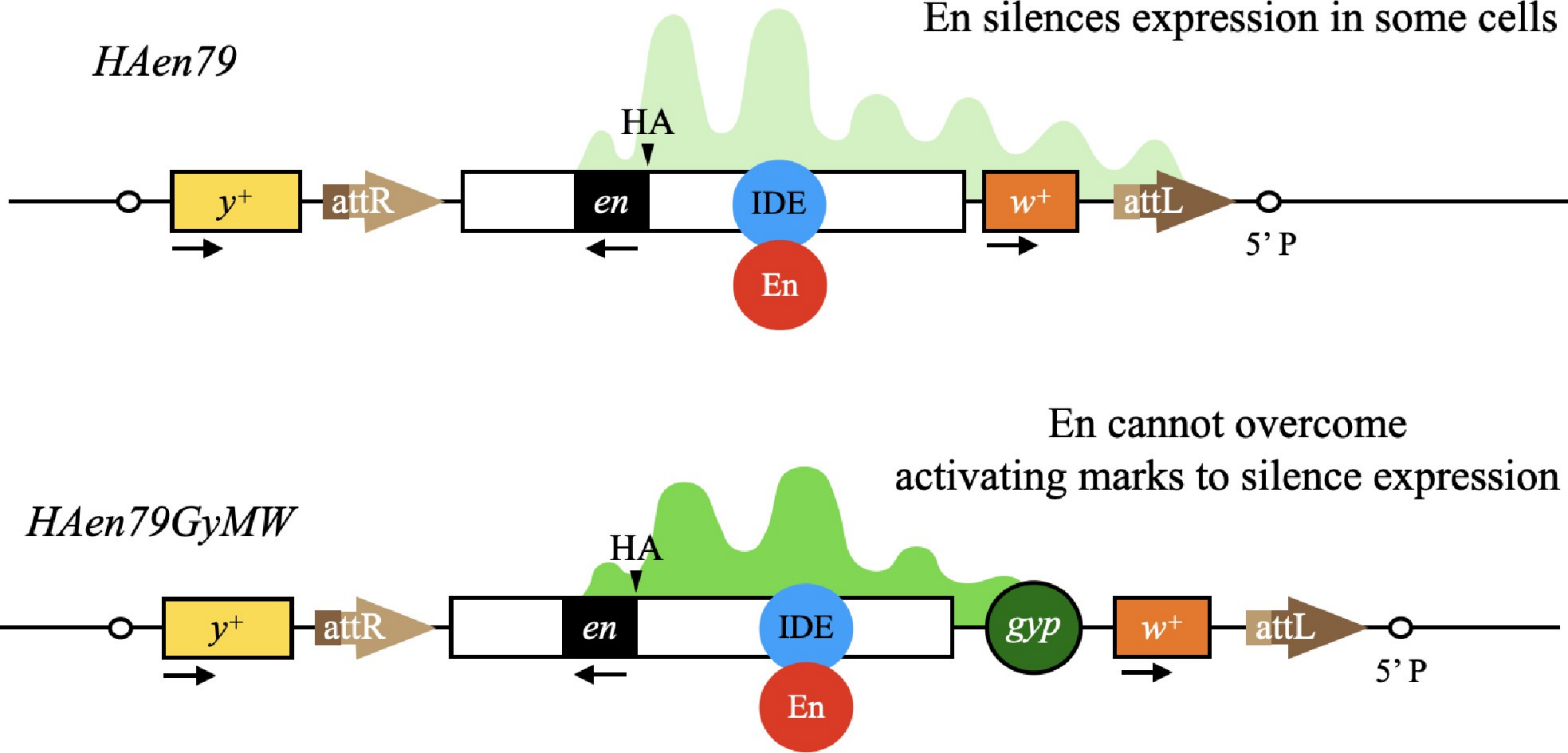
A model of how the gypsy boundary may increase the function of *HAen79*. The En protein (red circle) binds to an imaginal disc enhancer (IDE) in *HAen79* with (bottom) or without (top) a gypsy boundary inserted between *en* and *w+*. In this model, active chromatin marks (denoted by bright green peaks) cover the region upstream of the *en* transcription unit, spreading over the *w+* gene when a boundary is not present. The strength of activation (denoted by brighter green) is increased when the boundary is added. An alternative model is described in the discussion.

We were surprised that the activity of *HAen79GyW* is comparable to *HAen79GyB* for both viability as a heterozygote and HA-en expression in a wild-type background. Our previous data strongly suggested that all of the wing disc enhancers for expression in the posterior compartment are located upstream of the *en* transcription unit [12]. In addition, all of the Polycomb response elements (PREs) present in *HAen79* are located upstream of the *en* transcription unit [20,27]. Our data show that without the boundary between *en* and *w*, the chromatin-regulated imaginal disc enhancers can activate the flanking *w* gene. We suggest that this makes the overall stability of the ON state weaker and subject to repression by the endogenous En protein (see model Fig 7). In this model, we envision that active chromatin marks only cover the region upstream and within the *en* coding region in cells of the posterior compartment of imaginal discs. At the *Ubx* locus, only regulatory regions that are in use are covered with an active mark, H3K27ac; the rest of the regulatory DNA remains inactive chromatin and is covered by H3K27me3 [36]. There are no embryonic stripe enhancers or IDEs for the posterior compartment located downstream of the *en* transcription unit and we suggest that the active marks only spread in one direction: towards the *w* gene. This could explain why *y* is not expressed in the posterior compartment in *HAen79* wing discs. We propose that blocking the spreading of the active mark stabilizes the ON transcriptional state and lets it overcome repression by the En protein (Fig 7). The biological function of En binding to its own regulatory DNA might be to modulate its own expression levels which are not uniform in the posterior compartment.

Finally, we examined the stability of Polycomb repression of the *HAen79* transgenes with and without the gypsy boundaries. We previously showed that either removing the *en* PREs from *HAen79* or putting the *HAen79* transgene in a *ph-p*^*410*^ background (which reduces the amount of the PcG protein Polyhomeotic) leads to flies with disrupted abdomens due to mis-expressed En [27]. Adding a gypsy element to either one or both sides of *HAen79* reduces the abdominal phenotype seen in *ph-p*^*410*^ flies (S5 Fig). The exact phenotype was variable and similar in both *ph-p*^*410*^*; HAen79GyW* and *ph-p*^*410*^*; HAen79GyB* flies. The equivalent phenotypes obtained by flanking only one or both sides of *HAen79* with gypsy boundaries was surprising to us because H3K27me3 spreads in both directions past the *HAen79* ends into flanking DNA [27]. We suggest that the enhancers necessary for the abdominal phenotype are located upstream of the *en* promoter where both the constitutive and tissue-specific PREs are present [20], creating a stable H3K27me3 domain in the upstream DNA.

## Discussion

Here we have shown that the activity of two *en* imaginal disc enhancers is dependent on the chromosomal context. En is normally expressed in the posterior compartment in imaginal discs. When cloned into small transgenes, these enhancers induce expression mainly in the anterior compartment. En binds to both these enhancers and may act directly to repress their expression in transgenes. Deleting either enhancer from a 79kb-HA-en transgene caused defects in expression of HA-en in the posterior compartment and decreased its ability to function as the sole source of En. The same deletions in the endogenous *invΔ33* locus did not cause any obvious phenotypes. Inserting a gypsy boundary between the upstream En DNA and the mini-*white* gene in *HAen79* increases its activity, allowing it to rescue as a single copy, and increasing the stability of its expression when combined with a mutation in the PcG gene *ph*. Adding an additional gypsy boundary between the downstream *en* DNA and the mini-*yellow* gene did not increase the activity or stability of the transgene to a *ph* mutation further. In both *invΔ33* and *HAen79*, the *inv* PREs are missing, while the enhancers for expression in the posterior compartment of the wing disc, the precursors of the adult abdomen, and the constitutive and tissue-specific PREs are all located upstream of the *en* transcription unit (to the right of *en* in Fig 2 map). We therefore propose that by inserting gypsy to generate *HAen79GyW*, we block both the spreading of H3K27me3 and the enhancers from acting on mini-*white* and stabilize both the ON and the OFF transcriptional states; the other boundary is not important. Finally, deleting two imaginal disc enhancers from the *invΔ33* locus led to variegated En expression, suggesting that these enhancers are chromatin regulated and that additional imaginal disc enhancers exist somewhere in the *inv-en* domain.

### En represses its own expression

Previous studies have shown that overexpression of En can silence the expression of the En gene in wing discs [25,26]. Here we show that wildtype levels of Inv and En repress the expression of reporter genes that contain either fragment O or S in the posterior compartment of the wing disc. Furthermore, HA-en expression from the *HAen79* transgene is variegated in the wing pouch of the disc in a wildtype background. Repression of the *HAen45* transgene is even stronger and is relieved by a 53bp deletion in the *en* coding region that produces no En protein, showing that the repression is protein-mediated, and not via an interaction of the transgene with the endogenous locus as has been seen for *spineless* [30] Removing the imaginal disc enhancers from *HAen79* increases its repression in a wildtype background, suggesting that the En protein represses more strongly when there are fewer IDEs. ChIP experiments indicate that the En protein binds to both the O and S fragments, providing evidence that the repression by En may be direct. En is also bound to other places in the *inv-en* domain, including the *en* PREs (Fig 3), and we suggest that En represses itself through many DNA fragments in its own locus.

### The *invΔ33* domain is more stable than the *HAen79* transgene

To compare the activity of the *HAen79* transgene with the endogenous *inv-en* domain, we deleted 33kb of *inv* DNA, creating the allele we call *invΔ33*. Deletion of fragments O, S, or SS2 from *invΔ33* (*invΔ33ΔO*, *invΔ33ΔS*, *invΔ33ΔSS2* or *HAinvΔ33ΔSS2* heterozygous to *inv-en* deletions) did not lead to any defects in viability. Further, aside from holding their wings out, an indication of loss of *inv* DNA [20] there were no wing defects in these flies. In contrast, deletion of fragments O, S, or SS2 from the *HAen79* transgene led to defects indicative of a loss of *en* function, including defects in wing veins in the posterior compartment and the formation of anterior bristles on the posterior margin of the wing. Flies with a single copy of *HAen79ΔO enΔ110* or *HAen79ΔS enΔ110* did not hatch and had leg defects, indicating that these enhancers are also important for leg development. These data show that the endogenous locus is more resilient than the transgene to deletion of enhancers. Thus, our transgene experiments allowed us to detect enhancer activities that would not have been evident if experiments were only conducted on the endogenous *en* locus.

Deletion of both fragments O and SS2 from *invΔ33* led to variegated expression of En with variable wing defects. This result tells us two things: (1) there are other imaginal disc enhancers located within *invΔ33*, and (2) the variegated pattern suggests that the imaginal disc enhancers are regulated by chromatin modifications set earlier in development. We suggest that there is a competition between the ON and OFF states. In the presence of all the imaginal disc enhancers, the memory of the ON transcription state is maintained in the posterior compartment, perhaps by the Trithorax group proteins, and in the OFF state, the Polycomb group proteins are the winners.

### Adding a gypsy boundary to one or both sides of the *HAen79* transgene improves its function

Unlike the endogenous *en* locus, the *HAen79* transgene does not have any boundary elements. We previously showed that the H3K27me3 domain that forms over this transgene extends into flanking DNA until it is stopped by transcribed genes [27]. We also showed that genes flanking the *HAen79* DNA are expressed in a subset of En-expressing cells in embryos. Here we show that the flanking genes mini-*white* and mini-*yellow* are also expressed in wing discs, albeit in different parts of the disc. We hypothesized that adding boundary elements to the ends of *HAen79* would restrict the enhancers to the HA-en gene, improving its function. Surprising to us, adding a gypsy boundary between *HAen79* and the mini-*white* gene had the same effect on its function as adding it to both sides of En (Fig 7 and Table 2). In our model (Fig 7) we suggest that active marks spread over the mini-*white* gene, decreasing the level of the active chromatin mark, weakening the activity of the IDE. An alternative explanation is that the boundary between the *en* sequences and mini-*white* blocks transcription of a transcript initiated at the 5’P element promoter located upstream of *en* in *HAen79* (Fig 6A). A recent paper showed that transcripts generated by the P-element promoter in the absence of the Homie boundary are highly processive and repress *even skipped* enhancer and promoter activities [37]. In this model, *en* enhancers would stimulate the expression of both the mini-*white* and the P-element promoter. Read-through transcription from the P-element promoter, through the mini-*white* gene, and perhaps even the *en* regulatory and promoter regions, could interfere with their activity. In this second model, when gypsy is inserted between *en* and mini-*white*, the P-element promoter is not stimulated in an *en*-like pattern, and read-through transcription does not destabilize *en* expression.

### Similarities between *Ubx* and *en* imaginal disc enhancers

The homeotic gene *Ubx* specifies the formation of the haltere and must be silenced in the wing disc to prevent wing to haltere transformations. Nevertheless, there is an imaginal disc enhancer (IDE) within the *Ubx* locus that, when included in a reporter construct, causes the reporter gene to be expressed in both the haltere and wing discs. Combining this IDE with an embryonic *Ubx* enhancer that sets the boundaries of reporter gene expression in the embryo leads to reporter expression only in the haltere disc [13–16]. The silencing of the IDE enhancer is due to the Polycomb group genes. As shown here, the En IDEs work in a similar way. Another similarity is that high levels of Ubx protein can silence its own expression [38–40]. In fact, Ubx represses *Ubx* through directly binding to an IDE [41]. Further, mutations in Ubx binding sites within this IDE caused a loss of repression of a reporter gene but had no detectable effect on *Ubx* expression when made in the endogenous locus [41]. Thus, *Ubx* autoregulation modulates its own expression level throughout the haltere disc, and En likely does the same in the posterior compartment of wing imaginal discs.

## Concluding remarks

En is essential for Drosophila development in both embryos and imaginal discs, and the genome has devoted a lot of DNA to ensure its correct expression. We previously showed that the constitutive PREs are not required for viability or H3K27me3 domain formation at the *inv-en* domain in the laboratory [16–20]. Here we show that two imaginal disc enhancers are also not required for viability in the laboratory. What is not captured in our papers is that these PRE or enhancer deletion lines are not completely wildtype and are susceptible to mutations elsewhere in the genome. When we first isolated the *HAen79ΔO* and *HAen79ΔS* transgenes, there was a second site mutation located at another place on the second chromosome that made the wing phenotypes of these flies much more severe in *en* mutant backgrounds. It is possible that *invΔ33ΔO* and *invΔ33ΔS* flies are also susceptible to second site mutations; we did not test this. Thus, as seen at other loci (reviewed in [5]), we suggest that the seemingly redundant *inv-en* enhancers and PREs impart a stability that ensures robust development and resiliency important for survival outside of the laboratory.

## Materials and methods

### Small transgenes

Fragments O (2R:7,435,274..7,439,183) and S (2R:7,448,809..7,451,645) were cloned in a vector that contains about 8kb of upstream *en* regulatory DNA, including the *en* promoter (fragment H, 2R:7415785–7423711, [12]) (S1 Fig). All genomic coordinates are in genome release dm5. Fragments S, SS1(2R:7,450,142..7,451,645), and SS2 (2R:7,448,809..7,450,141) were cloned into the vector *pBPGUw* using the procedures described [42].

### *CRISPR/Cas9* mutants

The following mutant strains were generated with *CRISPR/Cas9* technology: *enΔ110* [27], *invΔ33* [27], *invΔ33ΔO*, *invΔ33ΔS*, *invΔ33ΔSS2*, *invΔ33ΔOΔSS2*, *HAinvΔ33ΔSS2*, *HAen*, *HAen79GyMW*, and *HAen79GyB* (this paper). Genomic coordinates: *invΔ33*, 2R:7,353,764..7,386,881 [27], ΔO, 2R:7,435,274..7,439,183; ΔSS2, 2R:7,448,809..7,450,141. ΔS in *invΔ33ΔS* is not a simple deletion: it deletes 2 fragments, one is 2683 bp, 2R:7,448,714..7,451,396 that has a 49bp insert sequence of unknown origin at the 3’ end of the deletion, and a smaller 241bp deletion, 2R:7,451,535..7,451,775. Note that 138bp between these two fragments is present. For CRISPR target sequence design and cloning into the pU6-BbsI-gRNA(chiRNA) vector we followed the protocols in https://flycrispr.org. Repair plasmids were used to make all new mutant fly lines except *invΔ33ΔS*. The repair plasmids were either synthesized by Genscript Inc or by assembling PCR fragments with NEBuilder (New England Biolabs). Typical repair plasmids had homology arms of 500 bp to 1000bp, depending on the experiment. The cloning vector for repair plasmids was pUC57. The gRNA plasmids were mixed equally to get a total concentration of 1 μg/μl. When a repair plasmid was used, the repair plasmids were mixed with gRNA plasmids at 0.5 μg/μl. The plasmid mixture was injected (Rainbow Flies, Inc or BestGene, Inc) into the relevant host fly strain expressing Vasa-Cas9 [43]. Genotypes injected were *M{vas-Cas9*.*S}ZH-2A*; with an appropriate 2^nd^ chromosome: wildtype, *invΔ33*, *invΔ33ΔSS2*, *HAen79*, or *HAen79GyW*. Adult flies from the injected embryos (G0) were singly crossed to a stock with a second chromosome balancer, *yw; Sco/CyO*. After about a week at 25°, when larval progeny were present, DNA was prepared from single fertile G0 flies. PCR was done to detect the desired modification in the single G0 flies. From G0s that gave a strong PCR signal, 20 of its progeny (G1) were singly crossed to *yw; Sco/CyO*. PCR was done on each G1 fly. The G1 flies that gave the right PCR product were used to establish a fly stock. After the fly stock was established, PCR and DNA sequencing were done to verify the desired change. A more detailed *CRISPR/Cas9* protocol for the generation of *HAen79GyMW* is described below. For other experiments, detailed protocols are available upon request.

The fly strain *y*^*1*^ *M{vas-cas9*.*S}ZH-2A w*^*1118*^*; HAen79* was generated using standard genetic crosses. A gRNA target site, ggccggccgcgatcgcgccc, was identified between mini*-white* gene and the 5’ end of *en* DNA present in *HAen79*. A repair plasmid was made using NE Builder (New England Biolabs) that includes a 430bp gypsy insulator sequence [44] flanked by two 1kb homology arms. G0 and G1 flies were screened and a fly stock was established. PCR and DNA sequencing were done to verify the gypsy insertion and flanking sequences, resulting in the *HAen79GyMW* strain.

#### Tagging en with HA

A 27 bp DNA sequence, TACCCCTACGACGTCCCCGATTACGCC, that encodes the 9 amino acids HA tag was inserted into *en* coding sequence immediately downstream of its translation start codon ATG onto either a wildtype second chromosome (*HAen*) or *invΔ33ΔSS2* (*HAinvΔ33ΔSS2*).

### Immunostaining and RNA-FISH

Our antibody staining procedure for imaginal discs has been described previously [12]. The primary antibodies used were: guinea pig anti-Inv (1:5000, [12]), rabbit anti-EN (1:500, Santa Cruz Biotechnology, Inc.), anti-HA.11 (1:1000, clone 16B12, Biolegend), rabbit anti-Gal4 activation domain (1:1000, Millipore Sigma). Alexa Fluor secondary antibodies were used (1:1000, Invitrogen) and discs were mounted in Vectashield with DAPI (Vector Labs). Fluorescent RNA in situ hybridization was done with DIG-labeled probes for *white* and *yellow* with TSA Plus Fluorescence Kits (Akoya Biosciences) and used for in situ hybridization to imaginal discs as described [45]. For all experiments, at least 10 discs were examined, except where noted in the figure legends.

### Large transgenes

Generation of the *HAen45*, *HAen79*, and *HAinv84* transgenes and transgenic lines were previous described [12]. The transgenes *HAen79ΔS*, *HAen79ΔO* and *HAen79ΔSS2* were generated using recombineering to delete DNA using the *HAen79* plasmid as the starting construct (for procedures see [12]). Detailed protocols are available upon request. The coordinates of the deletions were the same as in *invΔ33*, except that ΔS is a simple deletion in *HAen79ΔS* (2R: 2R:7,448,809..7,451,645). These transgenes were inserted at the attP40 insertion site (on chr2L) and recombined with *enΔ110*, *en*^*E*^, or *en*^*X31*^ (on chr2R) to generate the chromosomes used to test the transgene’s function in the absence of endogenous *inv* and *en*. PCR was used to detect the presence of the transgene and the *en* mutations.

## Supporting information

S1 Fig Activity of enhancers O and S in reporter constructs. (A) Diagram of *en-lacZ* reporter construct used. The 8kb *en* fragment drives *lacZ* expression in embryos in stripes but there is no expression of *lacZ* in the imaginal discs (Fragment H in [12]). (B, C) Expression of ßgal and En (B) and ßgal (C) from *S-enlacZ* inserted at 3 different insertion sites. (D, E) *O-enlacZ* inserted at 3 different insertion sites. See Table 1 for the coordinates of *en* fragments used in these experiments. At least 10 discs were examined for each genotype and a representative disc is shown.
(TIF)

S2 Fig Fragments O and S drive expression in other discs. (A) DNA construct and ßgal staining of imaginal discs from third instar larvae showing that the O enhancer drives expression of lacZ in the eye (E) and leg (L) discs (construct from [12]). (B) Gal4 expression from SS2@attP2 (Fig 3) in the haltere (H) and third leg (L) disc. There is less expression of Gal4 in the posterior part of the leg disc. In this halter disc, Gal4 appear to be expressed in the entire disc but in other haltere discs it was reduced in the posterior compartment. See Table 1 for the coordinates of En fragments used in these experiments.
(TIF)

S3 Fig HA-en expression from large transgenes is repressed by wildtype levels of En. (A, B) Expression of HA-en in wing discs from *HAen45* in a wildtype background (A) and on a chromosome with *en*^*B86*^ (B). *en*^*B86*^ makes no En protein but expresses Inv in a wild-type pattern. ((B) is reprinted from [12]) (C, D) Expression of *HAen79@attP40* in a wildtype background (C) and when on the same chromosome as *enΔ110* (D). *enΔ110* deletes the entire *inv-en* domain so Inv is not present. The white arrow points to a place where HA-en is not present. (E) HA-en expression from the *HAen45* and *HAen79* transgenes at different insertion sites. (F) *HAen79stop* expresses a non-functional HA-en protein. All discs are homozygous for the indicated genotype. (G) En in a wildtype wing discs. At least 10 discs were examined for each genotype and a representative disc is shown.
(TIF)

S4 Fig *HAen79* transgenes are haploinsufficient. (A) Wings of flies homozygous for the indicated genotypes. Asterisk marks the crossvein where minor defects are seen. Arrows point to the presence of anterior margin bristles on the posterior wing margin. A wildtype wing is shown with a line separating the A (anterior) and P (posterior) compartments. (B) Wings from flies of the indicated genotypes. The blue arrow points to the missing wing vein. The black arrow points to anterior bristles on the posterior wing margin. (C) Ventral side of pharate adults of the genotypes shown. On the left, two legs are visible; the middle leg has been removed. The boxed region is the sex comb teeth (sc). The distal part of this leg is missing and the proximal part is misshapen. This leg phenotype was extreme but seen in many *HAen79ΔS enΔ110* or *HAen79ΔO enΔ110/enX31* pharates. (D) This pharate has an extended wing missing many veins and less severe leg defects. (E) wildtype leg. Arrow points to the sex comb teeth. The distal part of the leg is missing in (C). Image of wildtype leg is from [49].
(TIF)

S5 Fig Adding gypsy elements to HAen79 improves the phenotype of the abdomen in *ph-p*^*410*^ mutant males. *invΔ33*, *HAen79*, *HAen79GyB* ventral-lateral views; *HAen79GyW* ventral view. Part of a leg is also evident in *HAen79GyB*. Only one copy of the transgene is present in these genotypes. *invΔ33* is heterozygous with a wildtype chromosome. Adding gypsy on one or both sides of *HAen79* led to similar phenotypes in *ph-p*^*410*^ that were more like wildtype. The phenotype is due to mis-expression of En in the progenitors of the abdomen. At least 10 flies of each genotype was examined, and a representative abdomen is shown.
(TIF)
