# Supplementary figures and images for: The activity of engrailed imaginal disc enhancers is modulated epigenetically by chromatin and autoregulation
Source: PLoS Genet. 2023 Nov 15;19(11):e1010826. doi: 10.1371/journal.pgen.1010826 (PMC10686433; doi:10.1371/journal.pgen.1010826)

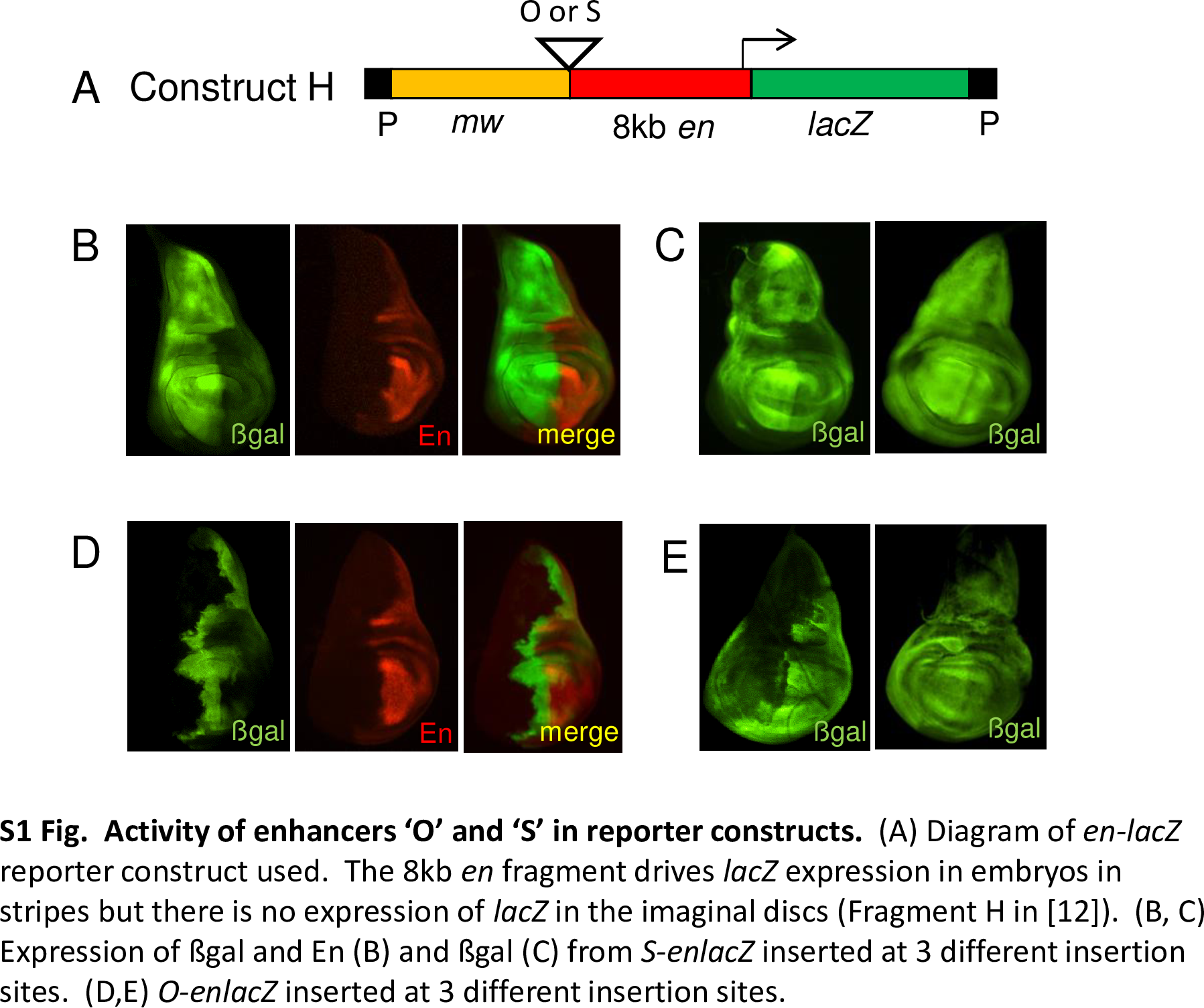

Supplement: S1 Fig — (A) Diagram of en-lacZ reporter construct used. The 8kb en fragment drives lacZ expression in embryos in stripes but there is no expression of lacZ in the imaginal discs (Fragment H in [12]). (B, C) Expression of ßgal and En (B) and ßgal (C) from S-enlacZ inserted at 3 different insertion sites. (D, E) O-enlacZ inserted at 3 different insertion sites. See Table 1 for the coordinates of en fragments used in these experiments. At least 10 discs were examined for each genotype and a representative disc is shown. (TIF) [file pgen.1010826.s001.tif]

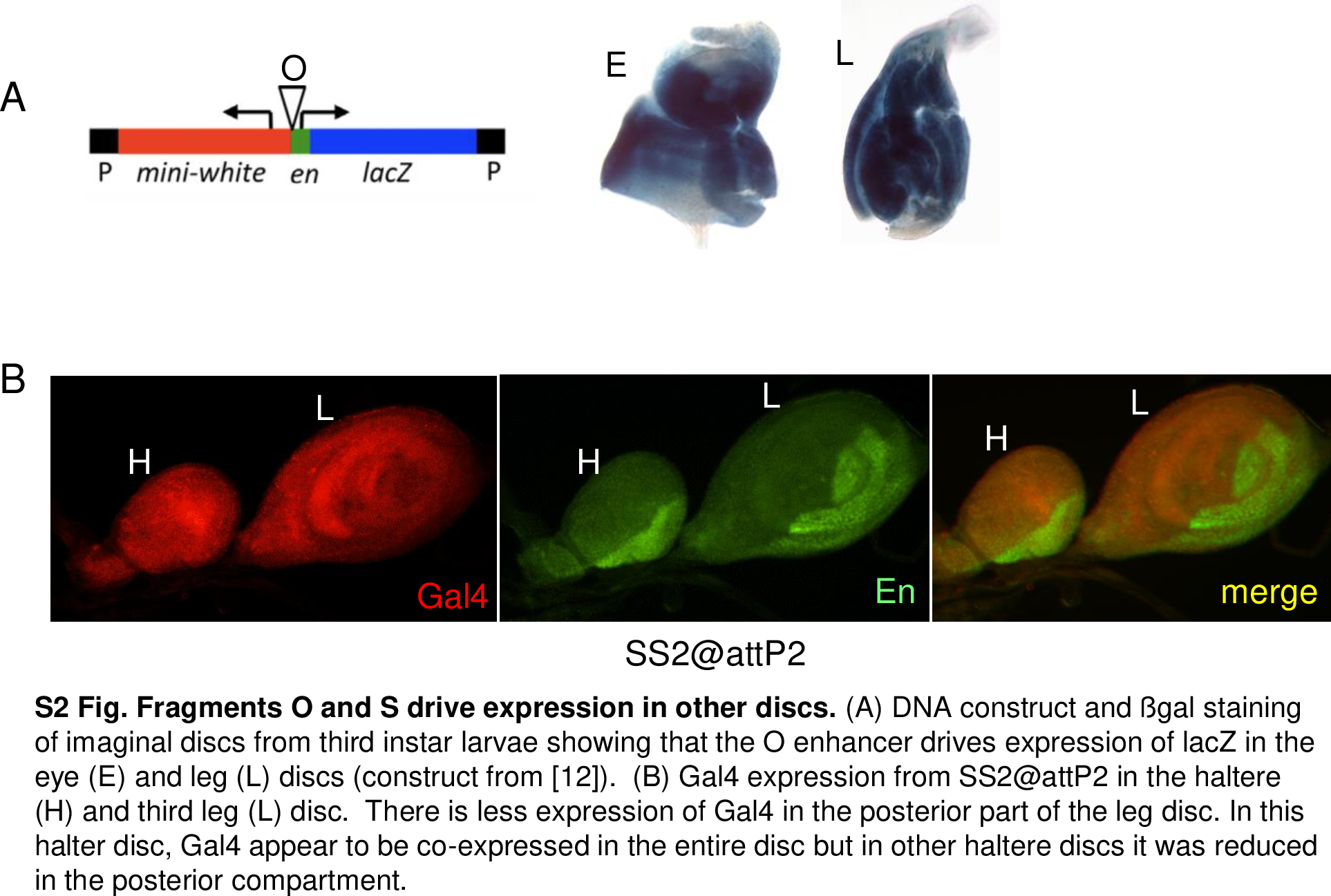

Supplement: S2 Fig — (A) DNA construct and ßgal staining of imaginal discs from third instar larvae showing that the O enhancer drives expression of lacZ in the eye (E) and leg (L) discs (construct from [12]). (B) Gal4 expression from SS2@attP2 (Fig 3) in the haltere (H) and third leg (L) disc. There is less expression of Gal4 in the posterior part of the leg disc. In this halter disc, Gal4 appear to be expressed in the entire disc but in other haltere discs it was reduced in the posterior compartment. See Table 1 for the coordinates of En fragments used in these experiments. (TIF) [file pgen.1010826.s002.tif]

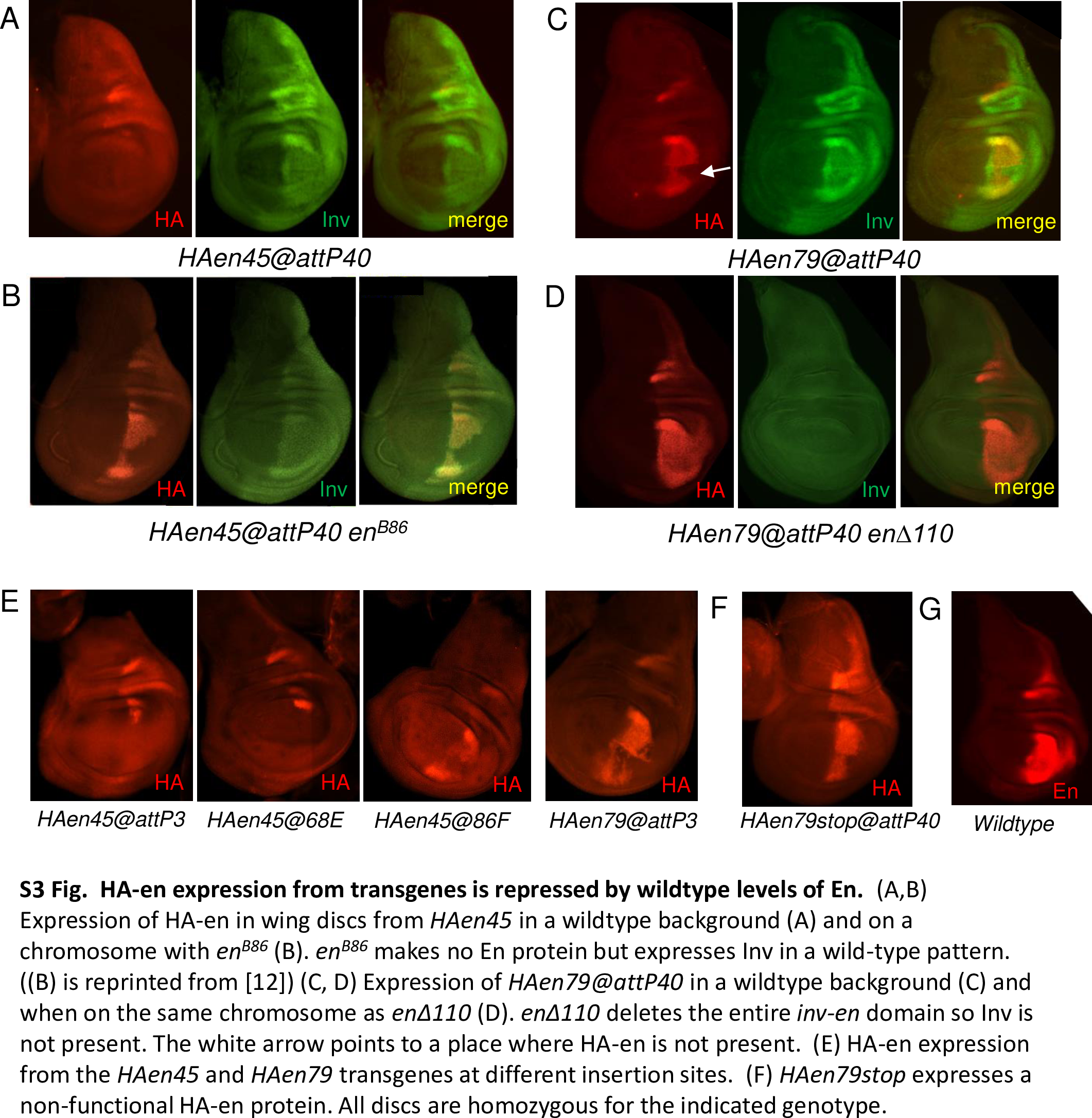

Supplement: S3 Fig — (A, B) Expression of HA-en in wing discs from HAen45 in a wildtype background (A) and on a chromosome with enB86 (B). enB86 makes no En protein but expresses Inv in a wild-type pattern. ((B) is reprinted from [12]) (C, D) Expression of HAen79@attP40 in a wildtype background (C) and when on the same chromosome as enΔ110 (D). enΔ110 deletes the entire inv-en domain so Inv is not present. The white arrow points to a place where HA-en is not present. (E) HA-en expression from the HAen45 and HAen79 transgenes at different insertion sites. (F) HAen79stop expresses a non-functional HA-en protein. All discs are homozygous for the indicated genotype. (G) En in a wildtype wing discs. At least 10 discs were examined for each genotype and a representative disc is shown. (TIF) [file pgen.1010826.s003.tif]

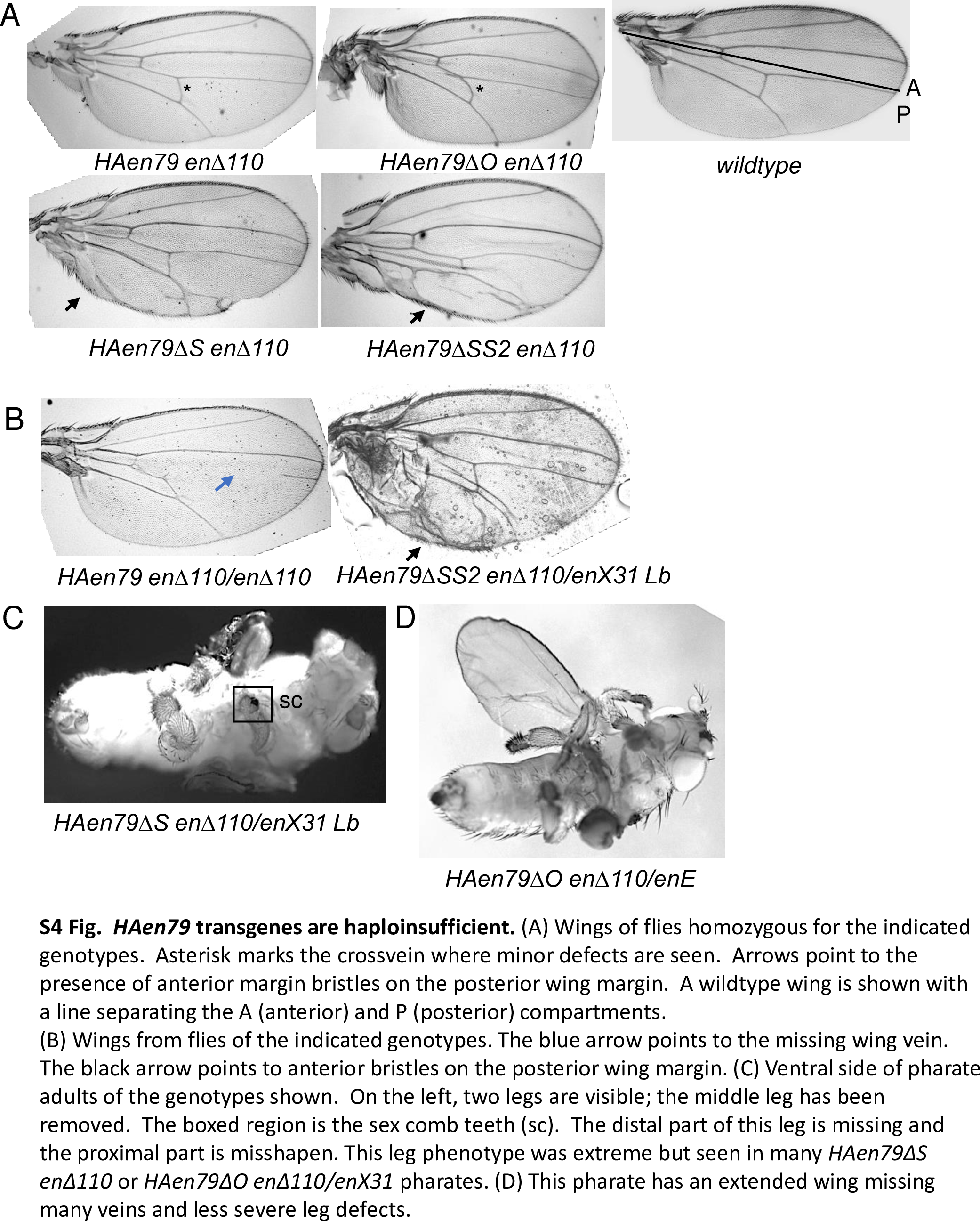

Supplement: S4 Fig — (A) Wings of flies homozygous for the indicated genotypes. Asterisk marks the crossvein where minor defects are seen. Arrows point to the presence of anterior margin bristles on the posterior wing margin. A wildtype wing is shown with a line separating the A (anterior) and P (posterior) compartments. (B) Wings from flies of the indicated genotypes. The blue arrow points to the missing wing vein. The black arrow points to anterior bristles on the posterior wing margin. (C) Ventral side of pharate adults of the genotypes shown. On the left, two legs are visible; the middle leg has been removed. The boxed region is the sex comb teeth (sc). The distal part of this leg is missing and the proximal part is misshapen. This leg phenotype was extreme but seen in many HAen79ΔS enΔ110 or HAen79ΔO enΔ110/enX31 pharates. (D) This pharate has an extended wing missing many veins and less severe leg defects. (E) wildtype leg. Arrow points to the sex comb teeth. The distal part of the leg is missing in (C). Image of wildtype leg is from [49]. (TIF) [file pgen.1010826.s004.tif]

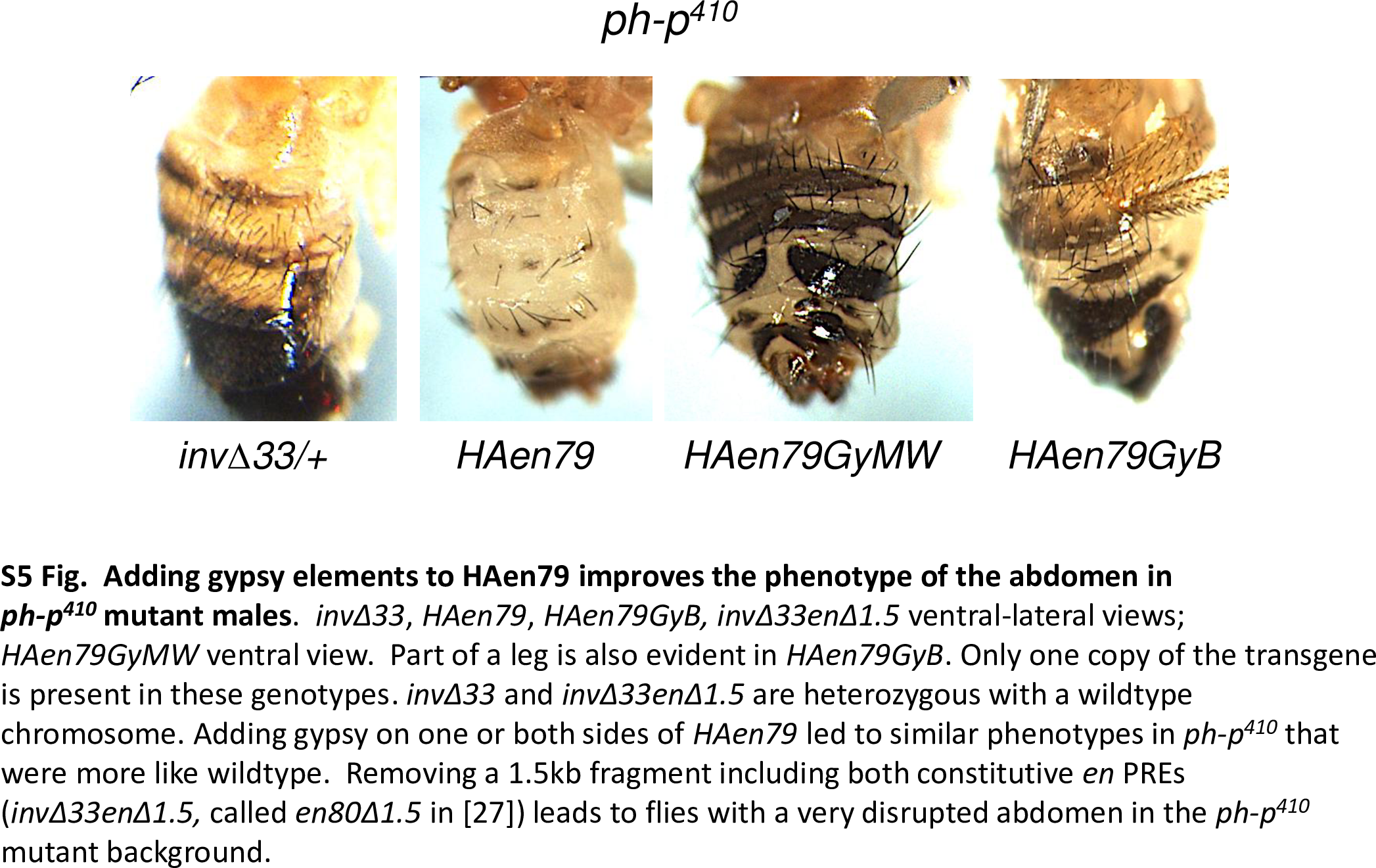

Supplement: S5 Fig — invΔ33, HAen79, HAen79GyB ventral-lateral views; HAen79GyW ventral view. Part of a leg is also evident in HAen79GyB. Only one copy of the transgene is present in these genotypes. invΔ33 is heterozygous with a wildtype chromosome. Adding gypsy on one or both sides of HAen79 led to similar phenotypes in ph-p410 that were more like wildtype. The phenotype is due to mis-expression of En in the progenitors of the abdomen. At least 10 flies of each genotype was examined, and a representative abdomen is shown. (TIF) [file pgen.1010826.s005.tif]
